# Metabolic Flux and Compartmentation Analysis in the Brain *In vivo*

**DOI:** 10.3389/fendo.2013.00156

**Published:** 2013-10-28

**Authors:** Bernard Lanz, Rolf Gruetter, João M. N. Duarte

**Affiliations:** ^1^Laboratory for Functional and Metabolic Imaging, Ecole Polytechnique Fédérale de Lausanne, Lausanne, Switzerland; ^2^Department of Radiology, University of Lausanne, Lausanne, Switzerland; ^3^Department of Radiology, University of Geneva, Geneva, Switzerland

**Keywords:** brain energy metabolism, neurotransmitter metabolism, neurotransmission, mathematical modeling, MRS

## Abstract

Through significant developments and progresses in the last two decades, *in vivo* localized nuclear magnetic resonance spectroscopy (MRS) became a method of choice to probe brain metabolic pathways in a non-invasive way. Beside the measurement of the total concentration of more than 20 metabolites, ^1^H MRS can be used to quantify the dynamics of substrate transport across the blood-brain barrier by varying the plasma substrate level. On the other hand, ^13^C MRS with the infusion of ^13^C-enriched substrates enables the characterization of brain oxidative metabolism and neurotransmission by incorporation of ^13^C in the different carbon positions of amino acid neurotransmitters. The quantitative determination of the biochemical reactions involved in these processes requires the use of appropriate metabolic models, whose level of details is strongly related to the amount of data accessible with *in vivo* MRS. In the present work, we present the different steps involved in the elaboration of a mathematical model of a given brain metabolic process and its application to the experimental data in order to extract quantitative brain metabolic rates. We review the recent advances in the localized measurement of brain glucose transport and compartmentalized brain energy metabolism, and how these reveal mechanistic details on glial support to glutamatergic and GABAergic neurons.

## Introduction

Localized magnetic resonance spectroscopy (MRS) is a powerful tool to investigate brain metabolism *in vivo*. MRS detection of ^1^H nuclei is widely employed because it takes advantage of this being the most sensitive nucleus in nuclear magnetic resonance (NMR). At high magnetic field it allows detection of a neurochemical profile of about 20 metabolites, particularly glucose, lactate, alanine, glutamate, glutamine, and aspartate, which are involved in energy metabolism and neurotransmission (1). Similarly, MRS of ^31^P provides a way of detecting non-invasively the phosphorus-containing metabolites, including the high energy phosphate compounds ATP and phosphocreatine, whose peaks can be used to determine the rate of creatine kinase that composes a brain’s energy buffering system [e.g., Ref. (2)]. NMR detects the non-radioactive, stable isotope ^13^C that occurs at a natural abundance of 1.1%, while the most abundant carbon isotope is ^12^C (98.9%), a NMR inactive nucleus. Nevertheless, the low natural abundance of ^13^C becomes an advantage when ^13^C-enriched substrates are administered and the rates of isotopic incorporation into specific carbon positions within different brain metabolites are dynamically detected (3–7). Upon employment of adequate mathematical models describing brain metabolism, this ^13^C incorporation rates can then be used to estimate fluxes through important metabolic pathways.

The application of dynamic ^13^C MRS to the study of brain energy metabolism and its coupling to neurotransmission has provided important insights on mechanisms supporting brain function but it also raised controversy on modeling approaches, metabolic assumptions in the models, and some extracted results. The significant developments on NMR technology over the last decade provided increase sensitivity and ^13^C MRS is now being performed with substantial improvements in spectral, spatial, and/or temporal resolution (8–11). This high quality data allowed determination of metabolic fluxes with better precision, but also made discrepancies between fitted curves and experimental data more apparent, suggesting that current models of brain metabolism lack a number of metabolic features necessary to fit experimental data (10, 12).

This review covers the approaches to design mathematical models of brain metabolism that allow quantification of metabolic fluxes from MRS data. Progress and controversies in the realm of brain metabolic modeling will be discussed. At last, we will discuss how ^13^C MRS data acquired at high-field support: (1) the role of glial metabolism in sustaining glutamatergic and GABAergic neurotransmission and (2) the coupling of the malate-aspartate shuttle with mitochondrial metabolism through the TCA cycle.

## Designing a Metabolic Model

An essential step to link the measured labeling time courses with biochemical quantities is the analysis with a metabolic model to derive quantitative metabolic fluxes. The most common models are the so-called multi-compartmental models.

A compartment is defined as an idealized store of molecules that exhibit the same behavior in a tracer experiment. Examples of compartments are the neuronal and glial compartments used in the ^13^C MRS modeling of brain glucose metabolism. In each compartment, molecules are physically or kinetically separated in metabolite pools. In the model, these are also called labeling pools and represent the major chemical intermediates involved in the metabolism of a particular substrate (e.g., glucose, acetate).

In ^13^C MRS experiments, for example, all isotopomers (i.e., isomers of isotope atoms) of a particular chemical species can appear in a metabolic pool, which in turn can be located in different compartments or physical environment (13). The number of labeling pools is reduced by lumping the chemical pools with similar characteristics and behaving identically into a limited set of pools.

The objectives of a metabolic model are the following:

1. identification of the structure of the system (pools and fluxes)
2. estimation of internal metabolic parameters
3. prediction of the response of the model to external factors

**Figure 1:**
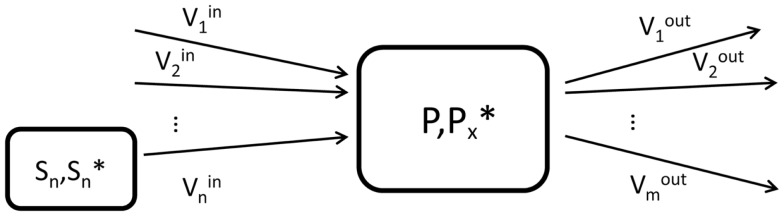
**Schematic view of the elementary unit (labeling pool) used in compartment modeling**. *P* is the total concentration of molecules in the labeling pool, while $P_{x}^{*}$ represents the concentration of labeled molecules at the molecular position *x*. In the general situation, the pool can have n influxes from *n* substrate pools *S_n_* and *m* outfluxes.

Briefly, a labeling pool that represents a molecule *P* labeled at a chemical position *x* is characterized by the total concentration *P* of the considered molecule, the concentration $P_{x}^{*}$ of those molecules labeled at the position *x* as well as by the set of inflows and outflows of the pool (Figure 1). The concentrations are usually expressed in micromoles per gram (μmol/g) of tissue, while the fluxes are given in micromoles per gram per minute (μmol/g/min).

For a given product *P*, the variation of total concentration of the product follows the mass balance equation (7, 13):

(1) $$\frac{dP\left(t\right)}{dt}=\sum_{i}^{n}V_{i}^{\text{in}}-\sum_{j}^{m}V_{j}^{\text{out}}$$

This equation is a mathematical expression of the fact that the variation of the quantity of molecules in the pool is the difference between what is entering the pool at a given time and what is exiting. In this general case, we consider *n* influxes and *m* effluxes. A simplification is often made by assuming that the biochemical system is at metabolic steady-state. In this case, the total concentration of the labeling pools as well as the metabolic fluxes between the pools are assumed constant over the duration of the measurement, which is a good approximation in many physiological conditions, for example for the glutamate and glutamine labeling pools in the case of [1-^13^C] or [1,6-^13^C_2_]glucose infusion (7, 14). At steady-state,

(2) $$\sum_{j}^{n}V_{i}^{\text{in}}-\sum_{j}^{m}V_{j}^{\text{out}}=0$$

The labeling dynamics of a metabolic pool *P* is governed by the isotope balance equation, which determines how the concentration of labeled molecules changes over time, as a function of the influxes and effluxes:

(3) $$\frac{dP_{x}^{*}\left(t\right)}{dt}=\sum_{i}^{n}V_{i}^{\mathrm{in}}\frac{S_{i}^{*}\left(t\right)}{\left[S_{i}\right]}-\sum_{j}^{m}V_{i}^{\mathrm{out}}\frac{P_{x}^{*}\left(t\right)}{\left[P\right]}$$

where we assumed a general elementary unit *P* of a metabolic model, as presented in Figure 1. The pool *P* has *i* influxes from *i* substrates *S_i_* (with total concentration [*S_i_*] and labeled concentration $S_{i}^{*}\left(t\right)$) and *j* outfluxes. The terms $S_{i}^{*}(t)/[S_{i}]$ and P*(*t*)/[*P*] can be understood in a probabilistic way as the probability that a molecule leaving the substrate pool *S_i_* is labeled and the probability that a molecule leaving the product pool is labeled, respectively. These dimensionless terms are called fractional enrichment (FE) and vary between 0 and 1.

### Model for a single metabolic pool

In many ^13^C labeling studies, the protocol of glucose infusion (bolus followed by a continuous infusion) is chosen so that the pyruvate enrichment has a shape close to a step function (7). The pyruvate enrichment is therefore approximated by:

(4) $$\begin{array}{l} \left\{\begin{array}{cc} \frac{S^{*}\left(t\right)}{S} & =0\;\mathrm{for}\;t<0\\ \frac{S^{*}\left(t\right)}{S} & =\mathrm{constant}\;\mathrm{for}\;t\geq 0 \end{array}\right. \end{array}$$

**Figure 2:**
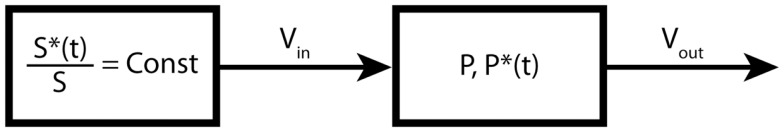
**One-product pool model**. In this example, the substrate pool is forced to maintain a constant fractional enrichment.

The time course of the precursor is called the input function and plays the role of a boundary condition of the system of ordinary differential equations. Since metabolic modeling is governed by linear differential equations, this will typically lead to exponential or multi-exponential solutions. If the input function is not a step function, then the exponential solutions are convoluted with the input function, which makes the system more complicated to analyze. In the case of bolus injections, which are widely used in positron emission tomography (PET) labeling experiments, a good knowledge of the exact shape of the input function is not always achievable, which can significantly affect the precision and accuracy of the derived fluxes (15). The mass balance equation for the one-product pool presented in Figure 2 is given by:

(5) $$\frac{dP\left(t\right)}{dt}=V_{\mathrm{in}}-V_{\mathrm{out}}$$

The labeling equation for the one-product pool is given by:

(6) $$\frac{dP^{*}\left(t\right)}{dt}=V_{\mathrm{in}}\frac{S^{*}\left(t\right)}{\left[S\right]}-V_{\mathrm{out}}\frac{P^{*}\left(t\right)}{\left[P\right]}$$

The general solution of P*(*t*) is:

(7) $$P^{*}\left(t\right)=\overset{t}{\underset{0}{\int }}V_{\mathrm{in}}\frac{S^{*}\left(t^{\prime }\right)}{\left[S\right]}\exp\left(\frac{V_{\mathrm{out}}}{\left[P\right]}\left(t^{\prime }-t\right)\right)dt^{\prime }$$

The integral expression in Eq. 7 is a convolution product in the sense of the Laplace transform. In fact, Eq. 7 is the convolution of the input function *S*^∗^(*t*′)/[*S*] with the impulse response of the system (in our case the metabolic system). With a steady-state enrichment of the precursor, i.e., with a step function as input, Eq. 7 simplifies to:

(8) $$P^{*}\left(t\right)=\overset{t}{\underset{0}{\int }}V_{\mathrm{in}}C_{\mathrm{FE}}\exp\left(\frac{V_{\mathrm{out}}}{\left[P\right]}\left(t^{\prime }-t\right)\right)dt^{\prime }$$

where *C*_FE_ is the constant fractional enrichment of the precursor.

Using the mass balance Eq. 5 in metabolic steady-state conditions (no net change in total concentration), we have *V*_in_ = *V*_out_. The solution for the fractional enrichment of the product pool is therefore given by:

(9) $$\frac{P^{*}\left(t\right)}{\left[P\right]}=C_{\mathrm{FE}}\left(1-\exp\left(-\frac{V_{\mathrm{out}}}{\left[P\right]}t\right)\right)$$

This is a typical expression for the labeling turnover of a pool with a step input function. In the field of linear differential systems, it is called the step response of the system. It is interesting to mention that the slope of this exponential curve at *t* = 0 is equal to *C*_FE_ *V*_out_/[*P*] (in this simple case, we have *V*_in_ = *V*_out_).

### Monte Carlo simulations for testing the reliability of flux estimations

Given the complexity of metabolic models, it is essential to ascertain that the analysis is robust and that the fit is not unstable, a situation that happens typically when a model is described with too many degrees of freedom compared to the available experimental data (7). Estimates of the standard deviation of the fitted parameters can be obtained from the fitting algorithm, by the calculation of the covariance matrix. The estimation of the covariance matrix by the calculation of the information matrix (16) is related to several assumptions on the variance of the data. However, for many MRS experiments, noise is not vanishingly small but frequently on the order of 10–30% of the steady-state labeling intensity. It was shown that the distribution of the fitted parameters can deviate strongly from the usually admitted normal distribution that is assumed by the fitting algorithms (6), which can lead to misinterpretation of the results and to false conclusions.

**Figure 3:**
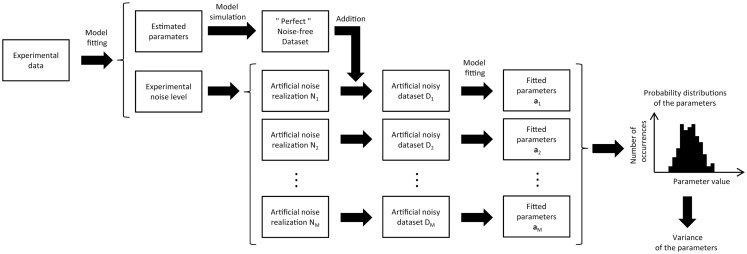
**Schematic view of the Monte Carlo analysis used in metabolic modeling to estimate the variance of the adjusted parameters**. The metabolic model is first fitted to the experimental data, giving an optimal parameter set and the related model turnover curves that best describe the data. From the fit residual, the noise level of the different turnover curves is estimated. Artificial datasets (typically several hundreds) are then generated by adding different noise realizations to the turnover curves obtained from the best fit. For each turnover curve, the artificial noise realizations are generated with the same noise level as in the corresponding experimental curves. Finally, the metabolic model is successively fitted to all the synthetic datasets, resulting in a distribution of fitted values for every free parameter of the model. This distribution characterizes the precision of each adjusted parameter, taking directly into account the experimental noise level.

One solution to the problem of non-negligible noise is the implementation of Monte Carlo simulations (17, 18). In principle, Monte Carlo approaches are simpler than analytical approaches but more computationally expensive. The objective of Monte Carlo simulations is to obtain the probability distribution of each estimated parameter. Briefly, the model is first fitted to the experimental data to obtain the most probable estimate of the model parameters, using non-linear regression. This set of parameters is then used to generate a “perfect” noise-free dataset, by simulating the enrichment curves with the metabolic model. This “perfect” dataset can be subtracted from the experimental data to estimate the noise level in each of the experimental labeling curves. In a second step, noise with the same distribution as the experimental noise is randomly added to each labeling curve of the “perfect” dataset to create a simulated noisy dataset. Finally, the model is fitted to this new artificial data to estimate the metabolic rates (Figure 3).

The process of generating and fitting artificial noisy datasets is repeated several hundred times to generate a list of simulated fitted parameters that are used to create a histogram of the distribution of the estimated parameter values. For each parameter, this histogram not only gives an estimation of its standard deviation but also information about the dissymmetry of its probability distribution (6, 7). The more Monte Carlo iterations are performed, the more accurate the probability distribution will be. In general, the initial guess for the estimated parameters used in the non-linear regression is also varied from one to the next Monte Carlo iteration. This avoids the optimization process converges to a local minimum.

In ^13^C MRS experiments, a set of ^13^C time courses is obtained from each subject and can be analyzed to obtain individual metabolic rates and their variation across the sample of the selected population. However, since it is known that the accuracy in flux estimation is inversely proportional to the noise level of experimental data, i.e., increases with reduction in the noise of ^13^C enrichment curves (19), ^13^C enrichment curves are often averaged across all subjects rather than fitting individual time courses. These averaged curves are then used for mathematical modeling and determination of metabolic fluxes. In this case, the variance across subjects can only be inferred from experimental ^13^C time courses, while the uncertainty of estimated fluxes is provided by Monte Carlo analyses.

## One-Compartment Model of Brain Energy Metabolism

In the past two decades, ^13^C MRS labeling studies raised a strong interest for the study of brain energy metabolism. Early studies were essentially undertaken by infusion of [1-^13^C]glucose (14, 20–22) and were analyzed with one-compartment models of brain metabolism.

**Figure 4:**
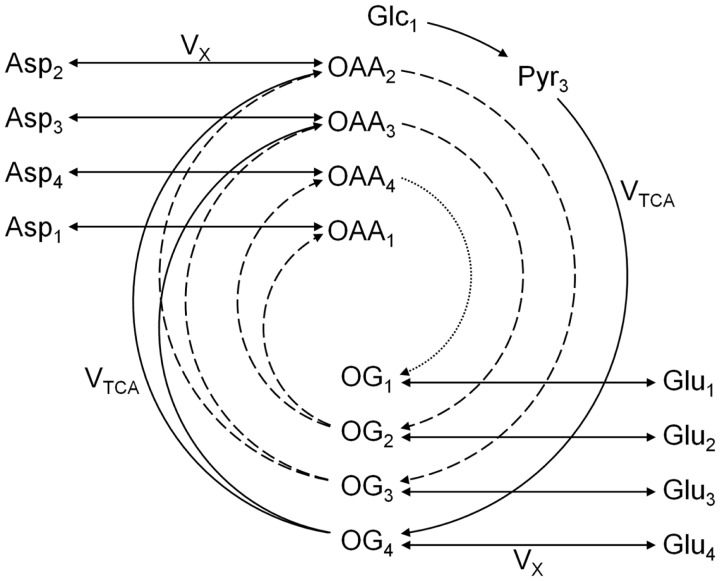
**Scheme of the one-compartment TCA cycle model**. The one-compartment model is characterized by the total TCA cycle flux *V*_TCA_ and the transmitochondrial flux *V_X_*. The splitting of the arrows after 2-oxoglutarate (OG) represents the symmetry at the succinate level. Glc, Pyr, OG, Glu, OAA, and Asp stand for glucose, pyruvate, 2-oxoglutarate, glutamate, oxaloacetate, and aspartate, respectively. The indexes represent the carbon positions that get labeled in each metabolite. The first, second, and third turns of the TCA cycle are represented by solid, dashed, and dotted lines, respectively. The model presented here corresponds to a [1-^13^C]glucose or [1,6-^13^C_2_]glucose infusion experiment.

The one-compartment model was the first metabolic model proposed to fit the glutamate C4 enrichment curves obtained following the infusion of [1-^13^C]glucose (20, 22). In this model, no distinction is made between neuronal and glial cells. Since most of glutamate is located in the neuronal compartment, the one-compartment model has been assumed to reflect primarily the neuronal TCA cycle rate. This model, depicted in Figure 4, enables the measurement of the overall (glial and neuronal) TCA cycle rate and the transmitochondrial flux *V_X_*, summarizing the glutamate to 2-oxoglutarate conversion and its transport across the mitochondrial membrane.

[1,6-^13^C_2_]Glucose is a widely used NMR tracer to probe mitochondrial metabolism. After transport across the blood-brain barrier (BBB), two molecules of [3-^13^C]pyruvate are generated from one molecule of labeled glucose through the glycolysis. When infusing [1-^13^C]glucose, only one molecule of [3-^13^C]pyruvate is produced, while the second pyruvate molecule generated by the glycolysis is unlabeled. The fate of the labeling from pyruvate C3 in the one-compartment model is depicted in Figure 4. Briefly, ^13^C from [1-^13^C] or [1,6-^13^C_2_]glucose enters both glial and neuronal TCA cycles at the position C4 of citrate. In the first turn of the TCA cycle, ^13^C reaches the position C4 of 2-oxoglutarate, which exchanges label with cytosolic glutamate. This transmitochondrial label exchange transfers label from the carbon position C4 of 2-oxoglutarate to the position C4 of glutamate. Due to the symmetry of the succinate molecule, the second turn of the TCA cycle brings half of the labeled carbons of the position C4 of 2-oxoglutarate to the position C3 of 2-oxoglutarate and the other half to the position C2 of 2-oxoglutarate. Through the transmitochondrial exchange, [3-^13^C]glutamate is formed from [3-^13^C]2-oxoglutarate and [2-^13^C]glutamate from [2-^13^C]2-oxoglutarate. In the third turn of the TCA cycle, half of the labeled carbons of the position C3 of 2-oxoglutarate reach the position C2 of the same molecule, while the other half remains at the position C3. At the same time, ^13^C from the position C2 of 2-oxoglutarate is transferred to the position C1, labeling further the position C1 of glutamate. The carboxyl position C1 of glutamate is usually not simultaneously measurable with the positions C4, C3, and C2 using ^13^C MRS, due to the large chemical shift of the C1 carbon position compared to the other resonances (23).

In some cases, the neurotransmission process is modeled in the one-compartment model in a simplified way by a glutamine exchange rate *V*_Gln_ (22, 24). Together with this flux, additional dilution of ^13^C enrichment at the level of glutamate from unlabeled glutamine was used to allow different fractional enrichments in glutamate C4 and C3 at steady-state, as observed experimentally. Although devoid of major effects in modeling ^13^C data from short experiments where isotopic steady-state is not reached, the inclusion of *V*_Gln_ and dilution from glutamine are crucial for reliable determination of *V*_TCA_ and *V_X_* in one-compartment modeling. This model was employed in recent studies to estimate fluxes from ^13^C curves of glutamate C4 and C3 (25–28).

Due to its intrinsic low sensitivity, ^13^C MRS only enables the measurement of metabolites that occur at sufficiently high concentration (typically >1 mM), most often allowing detection of the labeling positions of glutamate, glutamine, and aspartate. The pool size of oxaloacetate and 2-oxoglutarate is typically on the order of 0.1 μmol/g, while the pool sizes of glycolytic and TCA cycle intermediates are assumed to be small and without effect on the labeling dynamics of the observed amino acids (29). Thus, since the intermediate products of glycolysis and TCA cycle are present in too low concentrations to result in substantial delays in the labeling of the measured amino acids (i.e., small turnover times for these intermediates), the model can be simplified to retain only the labeling pools representing the MRS detectable amino acids and the intermediate pools at chemical branch points, such as oxaloacetate and 2-oxoglutarate. It was recently shown that the intermediate pools can be eliminated from the equations describing the one-compartment model, without affecting the dynamics of the glutamate uptake curves (30).

### The *V_X_*/*V*_TCA_ ratio

The labeling of the carbon positions in glutamate is the result of two processes: the TCA cycle (*V*_TCA_) and the transmitochondrial exchange (*V_X_*). These two fluxes are therefore intrinsically coupled to each other in the labeling equations describing glutamate ^13^C turnover. For the sake of argument, we analyze the relation between *V*_TCA_ and *V_X_* in the simple one-compartment model with exclusion of aspartate and glutamine (Figure 4). After simplification of the TCA cycle intermediates (30), the ^13^C labeling curves of the C4 and C3 positions of glutamate (Glu_4_ and Glu_3_) are given by:

(10) $$\begin{array}{ll} & \frac{d\mathrm{Gl}u_{4}\left(t\right)}{dt}=\frac{V_{X}\cdot V_{\mathrm{TCA}}}{V_{X}+V_{\mathrm{TCA}}}\left(\mathrm{FE}\left(\mathrm{Py}r_{3}\right)-\frac{\mathrm{Gl}u_{4}\left(t\right)}{\left[\mathrm{Glu}\right]}\right) \end{array}$$

(11) $$\begin{array}{llll} & \frac{d\mathrm{Gl}u_{3}\left(t\right)}{dt}=\frac{V_{X}\cdot V_{\mathrm{TCA}}}{\left(2V_{X}+V_{\mathrm{TCA}}\right)\left(V_{X}+V_{\text{TCA}}\right)}\; & & \;\\ & \;\times \left[V_{\text{TCA}}\text{ FE}\left({\text{Pyr}}_{3}\right)+V_{X}\frac{{\text{Glu}}_{4}\left(t\right)}{\left[\text{Glu}\right]}-\left(V_{X}+V_{\mathrm{TCA}}\right)\frac{{\mathrm{Glu}}_{3}\left(t\right)}{\left[\mathrm{Glu}\right]}\right] \end{array}$$

where FE(Pyr_3_) is the enrichment of the C3 position of pyruvate, the direct precursor of the TCA cycle, which is assumed to reach steady-state faster than the glutamate. [Glu] is the total glutamate concentration in the tissue (labeled and unlabeled).

Equation 10 highlights the fact that the labeling of the C4 position of glutamate is a combined effect of the TCA cycle activity and transmitochondrial transport, characterized by a composite flux *V*_gt_ (20, 30).

(12) $$V_{\mathrm{gt}}=\frac{V_{X}\cdot V_{\mathrm{TCA}}}{V_{X}+V_{\mathrm{TCA}}}$$

The solutions of these equations for a constant precursor enrichment FE(Pyr_3_) are given by:

(13) $$\begin{array}{ll} \mathrm{Gl}u_{4}\left(t\right) & =\mathrm{FE}\left(\mathrm{Py}r_{3}\right)\left[\mathrm{Glu}\right]\left(1-\exp\left(-t\frac{V_{\mathrm{gt}}}{\left[\mathrm{Glu}\right]}\right)\right) \end{array}$$

(14) $$\begin{array}{llll} \mathrm{Gl}u_{3}\left(t\right) & =\mathrm{FE}\left(\mathrm{Py}r_{3}\right)\left[\mathrm{Glu}\right]\left[1+\exp\left(-t\frac{V_{\text{gt}}}{\left[\mathrm{Glu}\right]}\right)\right.\; & & \;\\ & \;\left.-2\exp\left(-t\frac{V_{\text{gt}}}{\left[\text{Glu}\right]}\frac{\left(V_{X}+V_{\mathrm{TCA}}\right)}{\left(2V_{X}+V_{\text{TCA}}\right)}\right)\right] \end{array}$$

**Figure 5:**
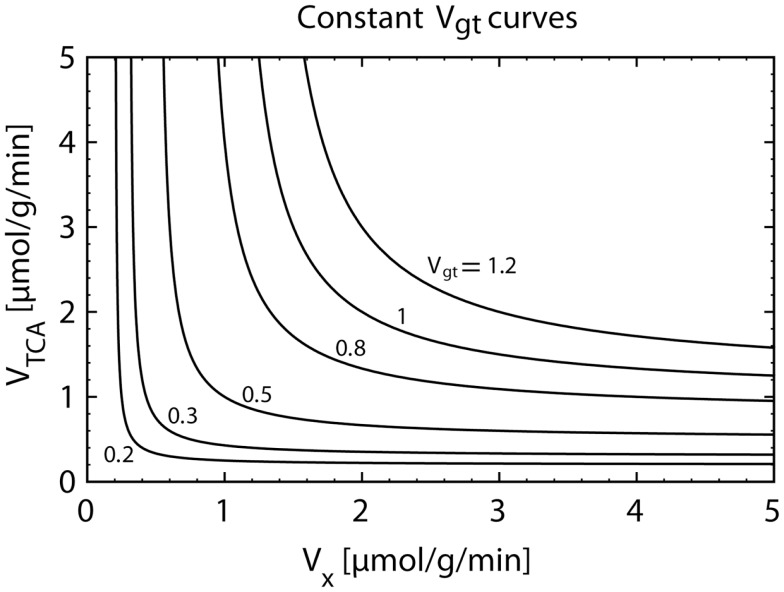
**Plot of the hyperbolic relationship between *V_X_* and *V*_TCA_ for a constant composite flux value *V*_gt_ (Eq. 12)**. The knowledge of *V*_gt_ alone only allows the calculation of *V*_TCA_ under an assumption for the value of *V_X_*. However, the magnitude of the assumed *V_X_* flux can have a strong impact on the estimated *V*_TCA_. When assuming *V_X_* ≫ *V*_TCA_, we have *V*_TCA_ = *V*_gt_. If *V_X_* is almost equal to *V*_TCA_, we obtain*V*_TCA_ ≅ 2⋅*V*_gt_.

The labeling equation of Glu_4_ only carries information on the value of *V*_gt_. Figure 5 illustrates the fact that an infinite number of pairs (*V*_TCA_, *V_X_*), distributed on a hyperbolic curve, result in the same value of *V*_gt_. Therefore, when fitting the Glu_4_ curve alone, the values of *V*_TCA_ and *V_X_* are not separately accessible.

However, the separate determination of *V*_TCA_ and *V_X_* is possible when fitting the model also to Glu_3_. This is directly related to the fact that the labeling equation of Glu_3_ does not depend only on *V*_gt_ (Eq. 14). *V*_TCA_ and *V_X_* have a distinct role in the labeling dynamics through the dilution term *V_X_*/(*V_X_* + *V*_TCA_).

The value of the *V_X_*/*V*_TCA_ ratio is still a matter of controversy. In early studies, the value of *V_X_* was reported to be much higher than the value of *V*_TCA_ (20, 22, 31, 32). In several following studies, it was therefore assumed that *V_X_* ≫ *V*_TCA_, which results in a simplification of the model, since in this case, *V*_gt_ = *V*_TCA_ (see Eq. 12). *V*_TCA_ could be therefore directly extracted from the fitting of the Glu_4_ curve. However, later *in vivo* and *in vitro* studies (9, 24, 29, 33–35) provided evidence that the value of *V_X_* is on the same order of magnitude as *V*_TCA_. In this context, using the assumption of a very large *V_X_* value leads to an underestimation of *V*_TCA_ by a factor of two, since for *V_X_* ≅ *V*_TCA_, we obtain *V*_gt_ ≅ *V*_TCA_/2.

**Figure 6:**
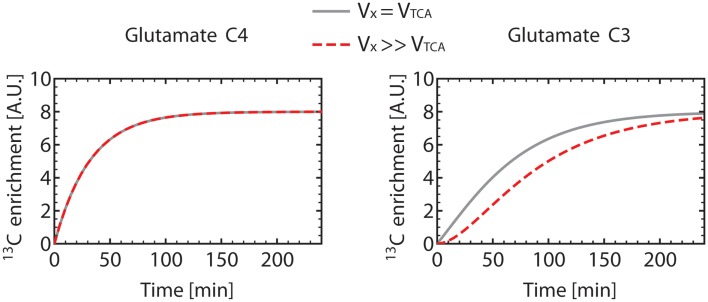
**Turnover curves of the carbon position C4 and C3 of glutamate as simulated using the one-compartment model (Figure 4) with a constant *V*_gt_**. For the gray turnover curves, *V_X_* was set equal to *V*_TCA_ (*V_X_* = *V*_TCA_ = 0.5 μmol/g/min), resulting in a non-zero slope of the glutamate C3 curve at *t* = 0. For the dashed red turnover curves, *V_X_* was set much larger (about 200 times) than *V*_TCA_ (*V_X_* = 50 μmol/g/min, *V*_TCA_ = 0.251 μmol/g/min), with the same value for *V*_gt_. This results in a sigmoidal-shaped glutamate C3 turnover curve, while the glutamate C4 curve remains unchanged. This observation illustrates the necessity to measure the C3 turnover curve to determine both *V_X_* and *V*_TCA_. In these simulations, dilution at the level of glutamate was not included, resulting in the same enrichment level for C4 and C3 at steady-state.

Figure 6 shows how Glu_3_ is affected by the value of the *V_X_*/*V*_TCA_ ratio. When *V_X_* ≫ *V*_TCA_, the initial slope of Glu_3_ is zero. When *V_X_* ≅ *V*_TCA_, the Glu_3_ curve loses its sigmoid shape, presents a non-zero initial slope and, furthermore, reached higher enrichment than for *V_X_* ≫ *V*_TCA_.

## Compartmentalized Brain Energy Metabolism Measured from Amino Acid Turnover

Metabolic compartmentation consists in the co-existence of separate pools of a given metabolite that are kinetically different and do not equilibrate rapidly with each other. Compartmentation of cerebral energy metabolism was initially identified by observing that certain radiolabeled tracers could lead to higher enrichment of glutamine than of its precursor glutamate (36, 37). In addition, different metabolic activities were observed in microdissected (38, 39) and cultured (40, 41) neurons and glial cells, and then glia-specific enzymes involved in intermediary metabolism were discovered in the brain tissue, namely glutamine synthetase (42, 43) and pyruvate carboxylase (44). The concept of exchange of metabolites between these compartments was also developed (45, 46) and resulted in the proposal of a glutamate-glutamine cycle linking glutamatergic neurons and astrocytes (47, 48).

Many studies followed and led to a growth of knowledge on the metabolic network underlying the interrelation between neurons and astrocytes. Nevertheless, we are still far from fully understanding the complex regulation of energy metabolism in the living brain. In this realm, the impressive development of localized ^13^C MRS *in vivo* since its first application to the head (49) has been much appreciated (4). Currently, the direct detection of ^13^C at high magnetic field provides a large amount of specific information regarding pathways of intermediary metabolism. In particular, ^13^C MRS is now able to quantify not only the incorporation of ^13^C into all aliphatic carbons of amino acids such as glutamate, GABA, glutamine, and aspartate, but also some multiplets resulting from the homonuclear coupling between adjacent carbons of these molecules, i.e., isotopomers (8, 50, 51). Alternatively, specific pathways can be addressed by providing certain labeling patterns to other carbons of glutamate and glutamine through administration of specifically labeled substrates (52). With this substantial increase in the amount of information from ^13^C MRS experiments at high magnetic field, higher accuracy has been achieved in the estimation of metabolic fluxes. However it is also becoming evident that state-of-the-art compartmental models of brain energy metabolism are unable to fully describe obtained experimental data (10, 12). Hereafter we describe and discuss the metabolic fluxes included in models of brain energy metabolism and suggest possible directions to improve the description of experimental data, namely by including sub-cellular compartments for particular metabolic pools.

### ^13^C MRS studies and modeling of metabolic compartmentation

**Figure 7:**
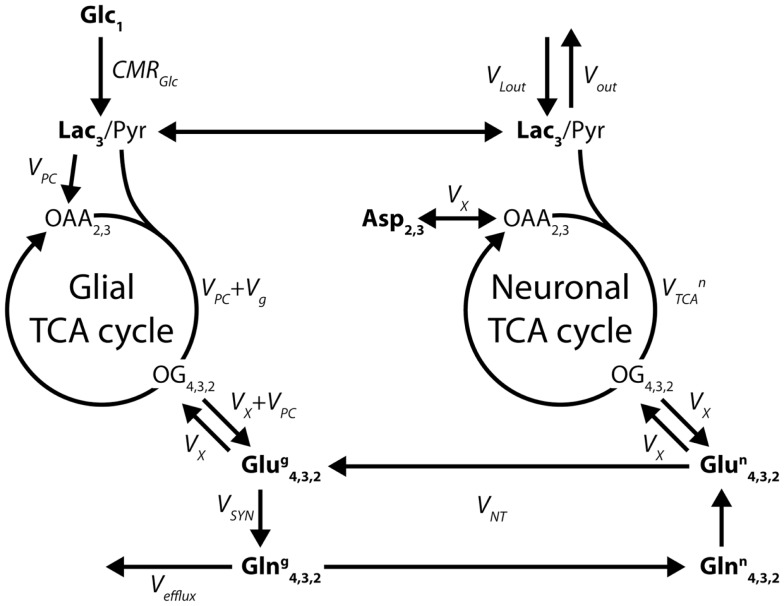
**Two-compartment model of compartmentalized brain metabolism, as proposed by Gruetter et al. (29)**. The model consists of the glial and neuronal TCA cycles, linked by the glutamate/glutamine cycle. Glc, glucose; Lac, lactate; Pyr, pyruvate; OAA, oxaloacetate; OG, oxoglutarate; Asp, aspartate; Glu, glutamate; Gln, glutamine. The system is characterized by the following fluxes: CMR_Glc_, cerebral metabolic rate of glucose; *V*_PC_, pyruvate carboxylase; *V*_g_ glial TCA cycle rate; *V_X_*, transmitochondrial exchange; $V_{\mathrm{TCA}}^{n}$, neuronal TCA cycle rate; *V*_SYN_, glutamine synthesis; *V*_NT_, apparent neurotransmission rate; *V*_efflux_, loss of glutamine from the glial compartment; *V*_out_ and *V*_Lout_, label dilution and exchange of lactate across the blood-brain barrier. The indexes represent the labeled carbon positions. Boldface indicates NMR-measurable metabolites.

When ^13^C glutamine was first detected *in vivo* in ^13^C glucose infusion studies (21, 53), it became natural to model the neuronal and glial TCA cycles and their interaction through the glutamate/glutamine cycle using a two-compartment model (29, 54), as shown in Figure 7.

Distribution of metabolic pools within these major compartments has been assumed based on data collected *in vitro*. It is well established that at least one small and one large glutamate pools exist in the glial and neuronal compartments respectively (55, 56). In contrast, glutamine has been mostly attributed to glia, where it is synthesized, while glutamate resides in neurons. Since ^13^C isotopes of the astrocyte-specific substrate acetate lead to labeling of glutamine to a greater extent than glutamate [e.g., Ref. (57)] this assumption seems to be valid. In a recent ^1^H-[^13^C] MRS study based on the infusion of glial-specific [2-^13^C]acetate (35), the glutamate pool distribution between the glial and neuronal compartments could be determined directly *in vivo* and supports the presence of a small glial glutamate pool accounting for about 5% of total glutamate.

However, ^13^C MRS measures the sum of all pools of a given metabolite. The detection of multiple compartments in the brain is only possible due to the presence of pyruvate carboxylation and glutamine synthesis in glia but not in neurons (42, 44) that leads to different label distribution in glutamate and glutamine. Pyruvate carboxylase is the main anaplerotic enzyme in the brain (58), leads to CO_2_ fixation and generates oxaloacetate that can be further used to generate new glutamate molecules. Pyruvate carboxylase brings ^13^C from [3-^13^C]pyruvate to the position C3 of oxaloacetate, which further labels the position C2 of 2-oxoglutarate and glial glutamate. On the other hand this reaction brings unlabeled ^12^C to the position C3 of glial glutamate. This glial dilution effect is one of the features that make it possible to distinguish glial and neuronal intermediary metabolism using glutamate and glutamine ^13^C time courses following infusion of [1-^13^C]- or [1,6-^13^C_2_]glucose. In addition, modeling of high-field ^13^C MRS data suggested that pyruvate pools may become differently labeled in neurons and astrocytes (10, 59, 60). In line with this, previous reports have frequently introduced glial and neuronal dilution fluxes at the level of lactate to account for this effect (61).

The glutamate-glutamine cycle is a major biochemical pathway *in vivo*, directly involved in the glutamatergic neurotransmission process (47, 48), and results from the compartmentation of glutamine metabolizing enzymes: glutamine synthetase is located exclusively in astrocytes (43), while the glutamate to glutamine conversion through phosphate activated glutaminase (PAG) occurs mostly in neurons (62). Furthermore, most of the glutamate is in the neurons, while glutamine is essentially located in the glial cells (56). Both glutamine and glutamate are 5-carbon chains differing by an amino group at the carbon position 5 of glutamine. In the glutamate-glutamine cycle, the carbon positions are maintained, which means that a carbon located at the position C4 of glutamate will reach the position C4 of glutamine and *vice versa*, and similarly for all positions of glutamate and glutamine.

The adjusted parameters of the two-compartment model (Figure 7) are the glial and neuronal TCA cycle fluxes *V*_g_ and $V_{\mathrm{TCA}}^{n}$, the transmitochondrial flux *V_X_* that describes the combined effect of glutamate dehydrogenase, aspartate transaminase, and transport across the mitochondrial membranes, the apparent neurotransmission flux *V*_NT_ and *V*_PC_, the rate of pyruvate carboxylation in the glia. A dilution *V*_out_ at the level of pyruvate is generally included to take into account the metabolism of other substrates, such as lactate or glycogen. Moreover, the glial acetyl-CoA, at the entrance of the TCA cycle, is diluted by alternative energetic fuels that glial cells can metabolize, such as acetate and fatty acids (63, 64). Glucose transport across the BBB is usually modeled using a Michaelis–Menten modeling approach (29), although more complex models have been described (65). In more recent *in vivo* studies, the two-compartment description of brain energy metabolism was further used for ^13^C labeling experiments using other substrates, such as [2-^13^C]acetate (35, 66, 67) or even adapted to describe the uptake curves measured by ^11^C positron emission when infusing [1-^11^C]acetate (68), bridging the gap between two major bioimaging modalities by proposing a common metabolic modeling approach.

Upon administration of ^13^C-enriched glucose, glutamine carbons become less enriched than those of glutamate and, to account for this discrepancy, dilution fluxes were placed in glutamine pools accounting for ^13^C loss by exchange with unlabeled pools (*V*_ex_), which can allow different compartment enrichments (33). Alternatively, other modeling studies assume different dilution fluxes at the level of pyruvate in each compartment, i.e., loss of labeling by exchange with unenriched pools of lactate (61). This accounts for possible ^13^C dilution by unlabeled brain glycogen or blood-born lactate or alanine.

There is now consensus that the interpretation of ^13^C incorporation curves from substrates into brain metabolites is never complete if mathematical models disregard metabolic compartmentation. This is particularly true when the fate of ^13^C is measured at high magnetic field where high spectral resolution allows for quantification of ^13^C in an increased number of amino acid carbons. Indeed, elegant simulations by Shestov et al. (19) demonstrated that increasing temporal resolution and decreasing noise level in ^13^C incorporation curves lead to increased accuracy in metabolic flux estimation. In addition, higher detail can be introduced in compartmentalized models of brain metabolism as more experimental curves are measured and for a longer period (19, 60).

### Sub-cellular compartmentation

Increased sensitivity in measuring ^13^C enrichment curves provides insight into sub-cellular compartmentation, which has been proposed in a plethora of studies *in vitro* (69–71). Sub-cellular compartmentation has been disregarded in most studies *in vivo*.

As explained above, carboxylation of [3-^13^C]pyruvate via PC labels C2 and dilutes C3 in glutamate/glutamine, resulting in observed relative enrichments of C4 > C2 > C3. In ^13^C MRS experiments at high magnetic field, with infusion of [1,6-^13^C_2_]glucose, glutamine enrichment in C2 was similar to that in C4 when approaching isotopic steady-state (9). The enrichment of glutamine C2 approaching that of the C4 carbon is consistent with high PC relative to PDH or with glial-specific dilution of acetyl-CoA, or a combination of both effects. Because metabolic modeling shows that such labeling patterns cannot be fully explained by pyruvate carboxylation, an additional glial-specific dilution flux *V*_dil_ has been introduced (9) to represent the utilization of glial-specific substrates that enter brain metabolism at the level of acetyl-CoA in astrocytes but not in neurons. The rate of *V*_dil_ was in the range of the rate of utilization of acetate *in vivo* (67, 72). This further ensures that pyruvate carboxylase and pyruvate dehydrogenase can carry different enrichment levels from pyruvate into the TCA cycle, as if distinct substrate sources would feed each pathway. This is also consistent with the presence of at least two distinct pyruvate pools in the glial compartment.

In cultured neurons, simultaneous incubation with [1,2-^13^C_2_]glucose and [3-^13^C]lactate revealed the existence of at least two cytosolic pools of pyruvate that do not equilibrate rapidly (73). While one of the pools was derived from glycolysis, the other was associated to lactate metabolism. Also in cultured astrocytes, labeling of alanine from ^13^C-enriched glucose has been observed to be lower than that of lactate, suggesting sub-cellular compartmentation of pyruvate (34, 74). One of the pyruvate pools could be specifically formed from TCA cycle intermediates. Indeed, in cultured astrocytes, the decarboxylation of malate by malic enzyme can represent a small fraction of the total pyruvate synthesis. This pyruvate formed via malic enzyme must then re-enter the TCA cycle to be oxidized, thus completing the pyruvate recycling pathway. This pathway has been proposed by ^13^C MRS of extracts from studies *in vitro* (34, 75–77) and *in vivo* (57, 78). This pathway is likely to be more active when cells need to dispose of glutamate and glutamine by oxidative degradation (77), which is an alternative to glutamine efflux from the brain that is generally modeled in ^13^C MRS data *in vivo* (*V*_efflux_, equivalent to *V*_PC_). The latter is however a recognized mechanism for ammonia disposal (79, 80).

However, if pyruvate recycling would be the main form of losing a four carbon intermediate from the TCA cycle to balance glutamate oxidation in the astrocyte, additional peaks would be observed in glutamate C4 in the ^13^C NMR spectrum. As discussed in Duarte et al. (9), while [1,6-^13^C_2_]glucose originates pyruvate labeled in C3, pyruvate recycling (coupled to glutamate oxidation) would generate pyruvate labeled in C2 or simultaneously in C2 and C3, which via pyruvate dehydrogenase originates 2-oxoglutarate and glutamate labeled in C5 or simultaneously C4 and C5. These were not detectable *in vivo* (9). Furthermore, labeling from pyruvate C2 is incorporated in lactate C2. In brain extracts at the end of the experiment, FE of brain lactate C2 was at least 20 times smaller than C3. Since this labeling patterns could also be observed in plasma lactate, probably resulting from peripheral metabolism rather than brain release, there is no convincing evidence that glutamate oxidation and pyruvate recycling are measurable *in vivo* upon infusion of [1,6-^13^C_2_]glucose (9, 12).

In addition, when steady-state of ^13^C enrichment was reached for carbons of glutamate and aspartate that have major pools in neurons, a significant and continuous increase in glutamine enrichment occurred (9, 12). This could be caused by increase of total glutamine concentration during glucose infusion, as was observed under hyperammonemia (81). However, brain glutamine concentration was not altered in similar experimental conditions (82, 83). While pyruvate formation from four carbon TCA cycle intermediates and further carboxylation could eventually explain such effect in C3 and C2, glutamine C4 only receives labeling from acetyl-CoA and thus should reach the same steady-state enrichment as carbons in the neuronal compartment (where PC is absent) and with which it exchanges via glutamate-glutamine cycle. This continuous increase in brain glutamine enrichment upon ^13^C-enriched glucose administration was observed in studies using dynamic ^13^C MRS *in vivo* in both rodents (9, 10, 12) and humans (84), and suggests that the existent mathematical models of brain metabolism are incomplete and may particularly benefit from inclusion of features like sub-cellular metabolic compartmentation or a multitude of astrocytes with heterogeneous metabolic rates. Accordingly, astrocyte morphology is compatible with the existence of different functional domains [reviewed by Pellerin and Magistretti (85)], there is mitochondrial heterogeneity and glutamine synthesis from multiple glial TCA cycles (69, 71) and, in cultured astrocytes, intracellular, and released glutamine were found to display distinct labeling patterns from metabolism of ^13^C-enriched glucose and lactate (70).

Upon this evidence supporting the existence of multiple glial glutamine pools, in our most recent ^13^C MRS study (12), two distinct pools of glutamine were modeled within the glial compartment, in addition to the pools in glutamatergic and GABAergic neurons. One of these glial glutamine pools was non-metabolizable and was in direct exchange with the other that was involved in the glutamate-glutamine cycle. Although this substantially improved fitting to the experimental data, the additional glutamine pool could as well be introduced in any other metabolic compartment and provide similar approximation to the experimental data. This was however a simple approach to tackle sub-cellular compartmentation in astrocytes. Although a more realistic model would rather include more than one glial compartment, the increasing number of unknown variables (fluxes) in the model would lead to increased uncertainty in flux estimation.

The inclusion of a vesicular glutamate pool in the neuronal compartment has also been proposed and seems to improve fitting of ^13^C multiplet data and increase accuracy in *V*_NT_ estimation (60). However, since this pool is actively involved in the glutamate-glutamine cycle, it does not allow for different curve shapes for the labeling of glutamate and glutamine.

## Metabolic Pathways Coupled to Brain Activity

Over the last two decades, systems neuroscience was revolutionized by blood oxygen level dependent (BOLD) functional magnetic resonance imaging (fMRI). BOLD fMRI measures the global hemodynamic response, i.e., changes in local cerebral blood flow, volume, and oxygenation, that are related to neuronal activity in the brain. Although the underlying mechanisms of cerebral hemodynamic control remain to be firmly established, the link between the modifications in neuronal activity and the observed hemodynamic response is known to require metabolic involvement of a multitude of cells, including astrocytes. In fact, due to the particular cytoarchitectural relation to both neurons (forming the tripartite synapse) and blood vessels (mostly arterioles and capillaries), astrocytes are ideally positioned to detect neuronal activity, transmit signals to neighboring vascular cells and regulate supply of energy substrates to neurons. Indeed, the link between cerebral activity and metabolic fluxes for production of energy has been suggested since early studies of brain energy metabolism (45).

### Neurotransmission and the rate of glucose oxidation

Above isoelectricity, glutamate-glutamine cycle increases ∼1:1 with glucose oxidation that has been proposed to occur mostly in neurons, thus providing coupling between functional neuroenergetics and glutamatergic neurotransmission (32, 86–88). Such analyses have however disregarded glial glucose oxidation, even though conversion of neurotransmitter glutamate to the electrophysiological inactive glutamine in astrocytes involves energy metabolism (89–91). Furthermore, this relation does not appear to stand for GABAergic neurotransmission, which relies on astrocytic oxidation of GABA (12, 87). Therefore, a more complete analysis of the metabolic fluxes involved in sustaining neurotransmission is required.

^1^H MRS has been used to report the time course of brain metabolites, mostly lactate, during focal activation. Increase in lactate concentration was reported upon sustained visual cortex stimulation first by Prichard et al. (92), suggesting a stimulation-induced increase in cerebral metabolic rate of glucose (CMR_Glc_). This observation was further confirmed in humans and rodents (93–97) and suggested to be associated to other metabolic modifications like a decrease in glucose concentration, as detected by *in vivo* ^1^H MRS (93, 98, 99), and in phosphocreatine *versus* inorganic phosphate in ^31^P MRS experiments (95, 100). In line with this, both glucose transport and consumption were found substantially reduced in the rat brain under isoelectricity (83, 101, 102).

With the increase in sensitivity and spectral resolution at high magnetic field, ^1^H MRS in the human visual cortex allowed detection of an increase in glutamate and a putative decrease in aspartate during stimulation, in addition to lactate and glucose modifications (97, 103), suggesting modifications in the flow through the malate-aspartate shuttle, possibly linked to adjustments in redox potential upon increased cerebral glucose consumption. Similar alterations of lactate, glutamate, and aspartate concentrations as well as increased alanine levels have been reported in the cortex of conscious rats upon sensory stimulation (104) and rats under light α-chloralose anesthesia upon trigeminal nerve stimulation (96). However, using *in vivo* ^1^H MRS in rats under α-chloralose anesthesia, Xu et al. (105) found that sensorial stimulation could lead to an increase in glutamine and a decrease in glutamate, *myo*-inositol, and phosphocreatine to creatine ratio in the focally activated primary sensory cortex, albeit the modifications in lactate and glucose concentrations upon cerebral activation were not detected. ^1^H-[^13^C] NMR spectroscopy studies in rodents upon infusion of ^13^C-enriched glucose measured an increased tricarboxylic acid cycle flux (*V*_TCA_) in focally activated primary sensory cortex during forepaw stimulation (106–108) that is certainly linked to the increased CMR_Glc_ and oxygen $\left(\mathrm{CM}R_{O_{2}}\right)$. Similar observations appeared in studies in humans (109). Altogether, these studies indicate that the BOLD fMRI signal-change is associated with an increase in oxidative metabolism.

### Glial metabolism supports neurotransmission

**Figure 8:**
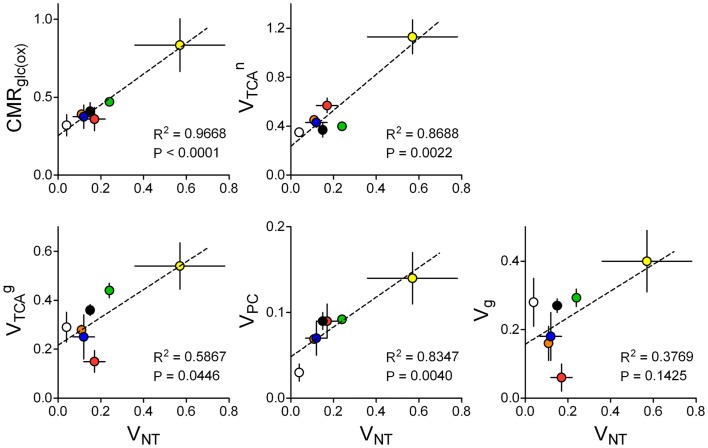
**Relation of estimated metabolic fluxes to glutamate/GABA-glutamine cycle, i.e., glutamatergic plus GABAergic neurotransmission (total VNT), across several studies using similar methodology in the rat or human brain: rat brain under pentobarbital-induced isoelectricity [white; Ref. (102)] and under light anesthesia [orange, blue, green, and black are data from Ref. (9, 10, 12, 35), respectively], human cortex [red; Ref. (29)], awake rat brain [yellow; Ref. (33)]**. Metabolic fluxes are shown in micromoles per gram per minute with associated SD. *P*-value for the slope and *R*^2^ are shown for each linear regression.

However, specific pathways of brain energy metabolism that support neurotransmission remain to be elucidated. Sibson et al. (86) suggested that in ^13^C MRS experiments, *V*_NT_ correlated with the neuronal fraction of CMR_Glc_ above isoelectricity, which has been confirmed in a large number of studies across different laboratories (32, 86–88). Other metabolic pathways may be linked to *V*_NT_ but remain to be analyzed. Figure 8 aims at elucidating how energy metabolism is related to both glutamatergic and GABAergic synaptic transmission. In contrast to previous analyses (88), now we exclusively selected ^13^C MRS studies in which metabolic modeling was performed with fully independent flux estimation (9, 10, 12, 29, 33, 35, 102). Interestingly this allowed identification of a correlation not only between *V*_NT_ and glucose oxidation (86, 88) but also between *V*_NT_ and *V*_PC_. Oxidative metabolism in neurons was correlated with *V*_NT_ only when GABAergic compartment is not included in the model. Correlation between glial TCA cycle and *V*_NT_ was noticeable in rats. Perhaps with additional experimental data sets from the human brain, we could depict a species-dependent relation of glial *V*_TCA_ to *V*_NT_.

Since its introduction, the astrocyte-neuron lactate shuttle model (89) has found additional confirmatory experimental data. Nevertheless it remains matter of debate, mostly because it considers that while neuronal metabolism is mainly oxidative, astrocytes exhibit a mainly glycolytic phenotype (85). In other words, glucose and oxygen are mostly consumed in astrocytes and neurons, respectively, and since brain hexokinase operates near it maximum rate assayed *in vivo* (65), only the neuronal TCA cycle can initially respond to the metabolic demand during increased brain activity [even though upon reduction of glucose-6-phosphate levels, there is loss of feedback inhibition and concomitant gain in hexokinase activity (110)].

The analysis in Figure 8 indicates that changes in glutamatergic neurotransmission, i.e., the glutamate-glutamine cycle, are as well coupled to glial pyruvate carboxylation and glial TCA cycle fluxes. This analysis agrees with simulations by Jolivet et al. (111) predicting that an important fraction of O_2_ utilization in astrocytes is dedicated to neurotransmission in the living brain. Furthermore, it is interesting to note that the four fluxes depicted in Figure 8 are significantly different from zero in the brain *in vivo* under isoelectricity (*V*_NT_ = 0), which would be associated to housekeeping tasks and other non-signaling brain processes. Recent estimations of energy budgets for the brain in awake state considered a 25 or 50% of the total energy budget for non-signaling tasks in either the cortical gray matter or the whole brain, respectively (112). If one considers that non-signaling and signaling energy metabolism takes place mostly in glia and neurons respectively, these data agrees with glial oxidative metabolism much larger than 25% of total energy consumption in the case of the whole brain. Thus, energy consumption for tasks unrelated to neurotransmission could account for up to ∼50% of total energy expenditure in the whole rat brain [discussed by Howarth et al. (112)], and may largely occur in glial cells.

Since only a fraction of $V_{\mathrm{TCA}}^{g}$ is associated with neurotransmission *V*_NT_, the remaining may be associated to housekeeping tasks. In line with this, there is no clear relation between *V*_g_ and *V*_NT_. *V*_g_ is a flux accounting for the difference between glial *V*_TCA_ and pyruvate carboxylase in the two-compartment model (see above). In the models where the GABAergic compartment is included and glial cells are oxidizing GABA, $V_{\text{g}}=V_{\text{TCA}}^{\text{g}}-V_{\text{PC}}-V_{\text{shunt}}^{\text{g}}$ [see Ref. (12)]. Since *V*_PC_ matches the efflux of glutamine and $V_{\text{shunt}}^{\text{g}}$ is equivalent to the shuttling of glutamine to the GABAergic neurons, *V*_g_ denotes the flux through glial pyruvate dehydrogenase corresponding to the complete oxidation of pyruvate. Therefore, in mathematical models designed as proposed in this manuscript, one can assume that pyruvate carboxylation and/or GABA oxidation are the main drive for the relation between $V_{\text{shunt}}^{\text{g}}$ and *V*_NT_.

Most recent publications reporting multi-compartmental models of brain energy metabolism constrained metabolic fluxes in astrocytes to those in neurons [discussed in Ref. (113)]. In particular, *V*_TCA_ in astrocytes and/or *V*_PC_ were systematically constrained as $V_{\text{PC}}=0.2\;V_{\text{GS}}$ and $V_{\text{TCA}}^{\text{total}}$ (61, 114). Other studies determined *V*_TCA_ in the astrocytic compartment from experiments upon [2-^13^C]acetate infusion and the obtained flux is used as constraint to the metabolic modeling of ^13^C-labeled glucose experiments for simultaneous determination of fluxes in glutamatergic and/or GABAergic neurons (115). In contrast, in studies that considered all metabolic fluxes as independent parameters, there was a fairly linear relation between pyruvate carboxylation rate (*V*_PC_) and the glutamate/GABA-glutamine cycle (Figure 8). In fact, the activity-induced increase in glial anaplerosis through *V*_PC_ is consistent with increased influx of bicarbonate into astrocytes upon neuronal release of K^+^ (116) that may stimulate pyruvate carboxylation (117). Flux through pyruvate carboxylation has been evaluated using [2-^13^C]glucose (for comparison with labeling from [1-^13^C]glucose see Ref. (54)). Interestingly, *V*_PC_ was not significantly altered in high neuronal activity upon by bicuculline-induced seizures (118), suggesting that pyruvate carboxylase and *de novo* glutamine synthesis are not required to support dysfunctional glutamatergic activity in this pathological condition.

Notably, in models that include GABAergic and glutamatergic neurons rather than a single neuronal compartment (12, 61, 119), correlation of total *V*_TCA_ in neurons with *V*_NT_ is not maintained because GABA-glutamine cycle relies on glial GABA oxidation in the TCA cycle [Figure 8, see also review by Hyder et al. (87)]. In line with this, nearly half of GABA produced in GABAergic neurons is further oxidized in astrocytes through the glial GABA shunt and half of GABA synthesis relies on glutamine provided by astrocytes (12), which is in accordance to observations in cultured cortical neurons supplied with ^13^C-enriched glutamine (120).

### Mitochondrial membrane transport is coupled to metabolic demand in neurons

Labeling of brain glutamate from a ^13^C-enriched oxidative substrate requires transfer of the label from the mitochondrial TCA cycle intermediate 2-oxoglutarate to cytosolic glutamate, which also sustains the transfer of reducing equivalents from cytosol to mitochondria. In the compartments where the GABA shunt occurs, i.e., glia and GABAergic neurons (12, 61), a substantial part of 2-oxoglutarate transamination to glutamate occurred with GABA, yielding succinic-semialdehyde that is further metabolized by the TCA cycles. Mitochondrial transport of ^13^C label in glia, which includes GABA transamination (in models with GABAergic compartment) and net glutamate synthesis, was much lower than the neuronal counterpart. This is in agreement with the finding that carriers exchanging aspartate and glutamate in the malate-aspartate shuttle are predominantly expressed in neurons rather than astrocytes (121–123). In contrast, comparable amounts of Aralar protein, the main mitochondrial carrier for aspartate/glutamate in the brain, were found in freshly isolated neurons and astrocytes (124), suggesting that the transfer of reducing equivalents across the mitochondrial membrane is not a limiting step for adaptation to altered metabolic or energetic demand in astrocytes. It is plausible that *V_X_* varies with increased metabolic activity as higher glycolysis requires transference of reducing equivalents into the mitochondria. However, this may be a small effect because of the inability to substantially increase hexokinase activity (discussed above).

As discussed above, in the absence of measurable ^13^C turnover curves for 2-oxoglutarate, the fluxes *V*_TCA_ and *V_X_* are intrinsically coupled (see Figure 5) and are of difficult determination when few ^13^C enrichment curves are taken for metabolic modeling. Although the rate of label transfer from 2-oxoglutarate to glutamate is frequently thought to occur at a more rapid rate than that of TCA cycle intermediate oxidation (22, 86, 125), metabolic modeling of ^13^C MRS data from humans (29) and rats (9, 10, 12, 33, 35, 102) with independent flux estimation found mitochondrial exchange fluxes on the order of the TCA cycle rate. In line with this, ^13^C-enriched 2-oxoglutarate but not glutamate was detected in the rat brain *in vivo* by ^13^C MRS upon infusion of hyperpolarized ^13^C-acetate (126). With further methodological development of these pioneering experiments, one can envisage that dynamic detection of labeling in both 2-oxoglutarate and glutamate will allow to reliably measure *V_X_*.

**Figure 9:**
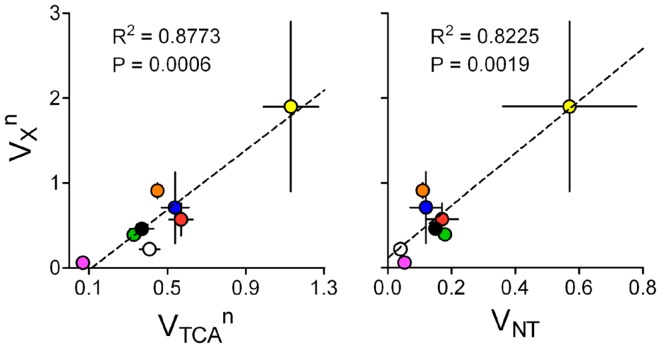
**$V_{X}^{n}$ and $V_{\mathrm{TCA}}^{n}$ are on the same order of magnitude and related (left)**. Accordingly, $V_{X}^{n}$ is also linearly related to glutamatergic or GABAergic *V*_NT_ (right). Data was collected from the same experiments as in Figure 8, which determined both fluxes in an independent manner: rat brain under pentobarbital-induced isoelectricity [white; Ref. (102)]; rat brain under light α-chloralose anesthesia [orange, blue, and black are from Ref. (9, 10, 35)]; rat brain under light α-chloralose anesthesia but data modeled with three metabolic compartments [pink/green are GABAergic/glutamatergic compartments in Ref. (12)]; human cortex [red; Ref. (29)]; awake rat brain [yellow; Ref. (33)]. Metabolic fluxes are shown in micromoles per gram per minute with associated SD. *P*-value for the slope and *R*^2^ are shown for each linear regression.

The fact that *V_X_* and *V*_TCA_ are on the same order of magnitude suggests that higher brain metabolic activity may require increased *V_X_*, especially in neurons. Interestingly, in mathematical models of brain energy metabolism where *V_X_* and *V*_TCA_ were determined independently from high resolution ^13^C MRS data, *V_X_* was related to *V*_TCA_ within the same compartment (Figure 9). This analysis was not performed for the glial compartment because most studies assumed identical *V_X_* for neurons and astrocytes. Moreover, when glial *V_X_* was independent, conversion of 2-oxoglutarate to glutamate is driven by *de novo* glutamine production, which is generally set to match *V*_PC_. Neuronal *V_X_* was also related to *V*_NT_ for the respective compartment, i.e., either glutamatergic or GABAergic neurons.

This subject requires further research, especially in conditions of high brain activity, since very few experimental data sets are available for analysis and most were acquired under anesthesia. In addition, since different models were generally used to estimate metabolic fluxes in different studies, this sort of analyses (Figures 8 and 9) should be considered cautiously. Employment of the same mathematical model to experiments under a range of brain activities and identical physiological conditions is required (86).

## Substrate Transport and Utilization

Proton MRS is emerging as an important tool for diagnosis and therapy monitoring as it provides biomarkers that offer fingerprints of neurological disorders in translational and preclinical neuroscience research (1). In addition, *in vivo* ^1^H MRS can be applied dynamically to evaluate cerebral cellular mechanisms that involve modification of metabolite concentrations, such as homeostasis disruption by pharmacological interventions (127) and substrate uptake and utilization (83).

Glucose transport kinetics as measured *in vivo* by ^1^H MRS has been mostly determined under steady-state conditions [e.g., Ref. (101, 128)]. Steady-state transport measurement from brain glucose content does not allow measuring glucose transport independently from glucose consumption but a ratio between the apparent maximum transport rate (*T*_max_) and the CMR_Glc_ is determined. Other studies determined glucose transport kinetics from variations of brain glucose content measured by ^1^H MRS upon a rapid change in plasma glucose concentration, which allowed quantifying both *T*_max_ and CMR_Glc_ (83, 129, 130). However, in these studies, different kinetic mechanisms have been defined for the glucose carriers at the BBB [discussed by Duarte and Gruetter (65)].

Initial modeling studies on glucose transport were based on the standard Michaelis–Menten kinetics to describe unidirectional fluxes across the membranes composing the BBB. Hexokinase, the rate-limiting step for glycolysis in the brain, operates close to saturation at physiological glucose levels. Thus, such standard Michaelis–Menten model predicted a maximum level for brain glucose, namely that brain glucose should be below 5 μmol/g for plasma glucose concentrations up to 30 mM.

However, brain glucose concentrations detected non-invasively by MRS are typically ∼9 μmol/g at plasma glucose concentrations of 30 mM in both humans and rodents [compared by Duarte et al. (128)]. At such high plasma glucose concentrations, glucose efflux from the brain is substantial and inhibits its own carrier-mediated uptake. Brain glucose concentrations above the *K*_M_ of GLUT1, the main glucose carrier at the BBB, imply that product formation is not unidirectional, i.e., the reverse reaction may proceed at significant rate. Glucose binding to the transporter at the abluminal membrane may partially inhibit the influx from the blood stream. When the product formation is not unidirectional, reversible Michaelis–Menten kinetics are applicable, which can be interpreted as reduced affinity for glucose influx, when substantial brain glucose is present. At steady-state, the reversible Michaelis–Menten model results in a linear relationship between brain and plasma glucose (102, 128, 131, 132). Dynamic ^13^C MRS studies with ^13^C-enriched glucose also demonstrated that transport of glucose into the brain can be predicted by the reversible model in rodents (9) and humans (29).

**Figure 10:**
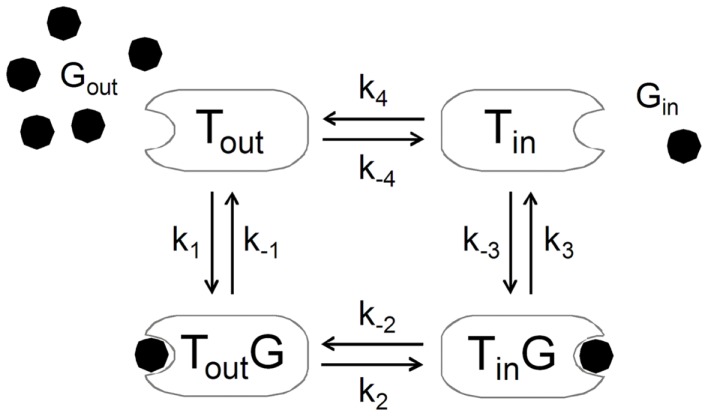
**The alternating-conformation kinetics of the glucose carrier**. In the absence of glucose (*G*_out_ or *G*_in_), the carrier can exist in two inter-converting isomers that are ready to bind glucose either outside (*T*_out_) or inside (*T*_in_) the membrane. When loaded, the carrier can also assume two isomeric forms favoring glucose release to the outer (*T*_out_*G*) or inner (*T*_in_*G*) side of the membrane. The rate constants *k*_1_ and *k*_−3_ define glucose binding while *k*_−1_ and *k*_3_ define its dissociation from the carrier. The rate constants *k*_2_ and *k*_−2_ or *k*_4_ and *k*_−4_ reflect the isomerization of the loaded or unloaded carrier.

In certain studies, deviation from this predicted linearity was observed at high glycemia (82, 133). This suggests that brain glucose can induce a certain degree of inhibition at the glucose carrier upon severe hyperglycemia, which is patent in experimental models of diabetes (82, 134, 135). This has been attributed to trans-acceleration or asymmetry that are not accounted for in Michaelis–Menten kinetics. Conversely, conformational four-state exchange kinetic models of solute carriers can simultaneous account for asymmetry, product inhibition, trans-acceleration, and multiple substrate competition (136, 137). Such kinetics was shown to efficiently describe brain glucose levels in multi-compartmental models of brain glucose transport (83, 128, 138). Unlike the reversible Michaelis–Menten model, this four-site exchange mechanism assumes that the free carrier after glucose release to the brain’s interstice is not conformationally the same that binds glucose outside, and that equilibrium exists between the two states of the unloaded carrier (Figure 10). The four-state conformational model predicts reverse glucose transport (and thus impediment of glucose uptake by the occupied carrier, like the reversible model) and that the presence of substantial amounts of glucose in the interstice prevents the carrier from acquiring a conformation that binds to glucose from plasma. In this case, for nearly symmetric carriers, the net transport can be expressed as:

(15) $$\nu_{t}=\frac{T_{\max}\left(G_{\text{plasma}}-G_{\text{brain}}\right)}{K_{t}+G_{\text{plasma}}+G_{\text{brain}}+\frac{G_{\text{plasma}}G_{\text{brain}}}{K_{\text{ii}}}}$$

where *K*_t_ is the apparent affinity constant and *K*_ii_ denotes the iso-inhibition constant that reflects the ability of glucose to inhibit the translocation of carrier isoforms between the two faces of the membrane. As previously demonstrated (128), the conformational model is equivalent to the standard Michaelis–Menten model for *K*_t_ close to K_ii_, and to the reversible Michaelis–Menten model at physiological glucose levels when K_ii_ largely exceeds *G*_plasma_. Since *K*_ii_ is indeed much larger than the concentrations of glucose typically observed in the brain (83, 128, 138), the transport mechanism based on the reversible Michaelis–Menten kinetics can fully describe glucose transport under normal physiological conditions. However, in metabolic conditions where high glucose concentrations are observed, namely under eventual uncontrolled diabetes (128, 135), the inhibition constant *K*_ii_ may be important in describing glucose transport at the BBB.

Magnetic resonance spectroscopy can be further extended to spectroscopic imaging with spatial resolution in the microliter range in rodents (139), being comparable to the spatial resolution of animal PET imaging with [^18^F]fluorodeoxyglucose but with the advantage of detecting glucose directly and, simultaneously, other neurochemicals involved in energy metabolism (1). Such methods can eventually be used to map regional glucose transport (140).

Although glucose is the main substrate for the brain, other compounds like ketone bodies, acetate, or lactate can be used as source of energy to maintain cerebral functions when glucose supply is limiting. Because they can be easily detected by ^1^H MRS (1), similar approaches could be used to determine their transport rates across the BBB. Although the potential of ^1^H MRS to non-invasively evaluate homeostasis of substrates other than glucose remain to be investigated, the measurement of the brain transport kinetics for lactate or acetate were accomplished by ^13^C MRS upon infusion of ^13^C-enriched tracers in rodents (67, 72) and humans (141).

## Glycogen Metabolism

Brain glycogen levels exceed those of glucose and are measurable in a non-invasive way by localized ^13^C MRS after administration of ^13^C-enriched glucose (142–144). In the adult brain, glycogen is primarily located in glial cells (145–148) as the glycogen synthesis machinery is physiologically inactive and glycogen phosphorylase nearly absent in neurons (149–151).

In the rat brain *in vivo*, glycogen concentration was determined to range from 3 to 6 μmol/g (144, 152–154). Its turnover time was found to lie between 5 and 10 h under physiological conditions (142, 144, 154, 155). Similar glycogen turnover time was reported in the brains of mice (156) and humans (143).

Regulation of brain glycogen levels is complex and not completely understood. Brain glycogen content was suggested to increase with circulating insulin and with brain glucose concentration (152, 153, 157–159). While glycogen content is increased under anesthesia (152, 160, 161), somatosensory stimulation increases glycogenolysis rate (162, 163) and thus reduces brain glycogen levels (164, 165). Although suggesting a direct role of glycogen metabolism in brain function, this was not observed under visual stimulation in both rodents (165) and humans (143). In the mouse hippocampus, glycogen was suggested to be involved in memory processing, being essential for long-term but not short-term memory formation (166).

Brain glycogen decreases upon insulin-induced hypoglycemia (152, 167, 168) due to enhanced glycogenolysis rate (157, 169, 170). It has been also proposed that recurrent hypoglycemia leads to increased substrate transport through the BBB, as well as to glycogen “supercompensation,” in which the brain adapts to hypoglycemia by increasing glucose storage in the form of extra glycogen content (157, 168). Similarly, glycogen supercompensation was observed after depletion upon exhaustive exercise in several brain areas of the rat brain (171). This role for brain glycogen in buffering neuroglycopenia suggests its involvement in hypoglycemia unawareness, which is defined as impaired counter-regulatory hormonal responses to glycemia challenges and loss of the neurogenic (autonomic) warning symptoms of developing hypoglycemia. Unawareness of low blood glucose is eventually caused by recurrent hypoglycemia induced by intensive insulin therapy in diabetes, particularly type 1 diabetes mellitus. Glycogen supercompensation in the brain after episodes of hypoglycemia and its role in hypoglycemia unawareness have hitherto been matter of debate (133, 157, 167–169, 172, 173). However, a variety of inconsistent experimental protocols have been employed to tackle this question, and it is not excluded that regulation of glycogen levels is also affected by the duration of hypoglycemia insults or the glycemia levels in the immediate period after hypoglycemia.

In summary, although brain glycogen metabolism presents itself as having roles in glucose buffering upon limited glucose supply, in either physiological or pathological conditions, the exact mechanisms of glycogen metabolism regulation *in vivo* remain to be understood. Modeling of glycogen metabolism in the brain *in vivo* generally assume that the rates of binding and releasing glucosyl units from the glycogen molecule are equal and that all glycogen molecules display the same behavior independently of their size (142, 155, 169). The parameters extracted from such models are limited to glycogen concentration and turnover. However, due to its tight and complex regulation (174), study of glycogen metabolism *in vivo* may require an analysis with mathematical models including a more complete representation of the regulatory network, as has been done for other organs with metabolic control analysis [e.g., Ref. (175)].

## Conclusion

The recent developments in the field of *in vivo* dynamic MRS of brain metabolism have provided precious information on substrate transport and utilization as well as neurotransmission mechanisms. A quantitative interpretation of these data requires advanced metabolic modeling approaches based on the biochemical knowledge accumulated over several decades. The level of complexity of these mathematical models strongly depends on the amount of information accessible *in vivo* and limits the number and precision of measurable metabolic rates.

In this review, we explained and discussed the methodology applied in mathematical modeling of brain energy metabolism measured with dynamic MRS, the assumptions required for modeling, and the ways to estimate the robustness and adequacy of a model. Monte Carlo simulations proved to be a precious tool for this purpose. Although some metabolic flux values are still a matter of debate, compartmental modeling of brain metabolism of ^13^C-labeled energy substrates shined a new light on the understanding of neuronal and glial oxidative reactions and neurotransmission processes. Recent studies showed that substantial glial metabolism supports both glutamatergic and GABAergic neurotransmission. Even though the results obtained with two- and three-compartmental models in those works tend to come to a good agreement, some evidence supports the fact that the current models of brain energy metabolism fail to completely describe ^13^C MRS data.

The recent dynamic ^13^C isotopomer analysis using multiplets from homonuclear ^13^C coupling (10, 51) or the use of hyperpolarized ^13^C methods (59, 126) may help to solve several remaining questions concerning brain metabolic processes. Overall, the availability of high magnetic field NMR systems and the continuous improvements in the detection methods in both ^13^C and ^1^H MRS enable the non-invasive acquisition of metabolic data with a steadily increasing level of detail and precision, which will require improvement of current metabolic models but is also expected to provide new insight in the understanding of brain energy processes and brain function in the near future.

## Conflict of Interest Statement

The authors declare that the research was conducted in the absence of any commercial or financial relationships that could be construed as a potential conflict of interest.

## Abbreviations

ATP, adenosine-5′-triphosphate; FE, fractional enrichment; fMRI, functional magnetic resonance imaging; MRS, magnetic resonance spectroscopy; NMR, nuclear magnetic resonance; TCA, tricarboxylic acid.
